# High expression of IMPDH2 is associated with aggressive features and poor prognosis of primary nasopharyngeal carcinoma

**DOI:** 10.1038/s41598-017-00887-1

**Published:** 2017-04-07

**Authors:** Yi Xu, Zhousan Zheng, Ying Gao, Shiyu Duan, Cui Chen, Jian Rong, Kebing Wang, Miao Yun, Huiwen Weng, Sheng Ye, Jiaxing Zhang

**Affiliations:** 1 Department of Oncology, The First Affiliated Hospital, Sun Yat-Sen University, Guangzhou, 510080 China; 2 Sun Yat-sen University Cancer Center, State Key Laboratory of Oncology in South China, Collaborative Innovation Center for Cancer Medicine, Guangzhou, 510060 China; 3 Department of Pathology, Nanfang Hospital, Southern Medical University, Guangzhou, 510515 China; 4 Department of Pathology, School of Basic Medical Sciences, Southern Medical University, Guangzhou, 510515 China; 5 Department of Extracorporeal Circulation, The First Affiliated Hospital, Sun Yat-sen University, Guangzhou, 510080 China; 6 Department of Surgical Laboratory, The First Affiliated Hospital, Sun Yat-Sen University, Guangzhou, 510080 China; 7 Department of Ultrasound, Cancer Center, Sun Yat-Sen University, Guangzhou, 510060 China

## Abstract

Inosine monophosphate dehydrogenase type II (IMPDH2) has been shown to play critical roles in the development and progression of several human cancers. However, little is known about IMPDH2 expression and its clinical significance in nasopharyngeal carcinoma (NPC). Western blotting, qRT-PCR and immunohistochemistry were employed to evaluate IMPDH2 expression in NPC cell lines and tissues. In our study, elevated expression of IMPDH2 was observed at both the protein and mRNA levels in NPC cell lines than in NPEC2 Bmi-1. IMPDH2 protein expression was markedly higher in NPC tissues than in adjacent non-tumorous tissues. Moreover, IMPDH2 expression in NPC correlated with several clinicopathological parameters, including T classification (P = 0.023), TNM stage (P = 0.020), distant metastasis (P = 0.001) and death (P = 0.002). Further Cox regression analysis suggested that IMPDH2 expression was an independent prognostic factor for overall survival (P = 0.001) and disease-free survival (P < 0.001). In addition, stratified survival analysis showed that high expression of IMPDH2 could be a prognostic factor for NPC patients with TNM stage I/II (OS: P = 0.012; DMFS: P = 0.007), TNM stage III/IV (OS: P = 0.028; DMFS: P = 0.020). Our study demonstrates IMPDH2 may be served as an independent prognostic biomarker for NPC patients, in which high IMPDH expression suggests poor prognosis of NPC patients.

## Introduction

Nasopharyngeal carcinoma (NPC) is one of the most prevalent human head and neck malignancies in Southern China, Southeast Asia and North Africa^1–3^. Due to its nonspecific symptoms in the early stage, the majority of NPC patients are present with advanced stage disease when first diagnosed. To date, the clinical TNM staging system for NPC is most commonly used to evaluate prognosis. Intriguingly, the prognosis of NPC patients with the same stage of disease often varies^4, 5^. Therefore, a large number of investigations on NPCs have focused on the discovery of specific biomarkers which could be utilized as more effective prognostic predictors. To date, however, highly sensitive and specific prognostic biomarkers remain unavailable for early diagnosis and individualized treatment of patients with NPC.

Inosine monophosphate dehydrogenase (IMPDH) is a rate-limiting enzyme which catalyzes a crucial step in the de novo biosynthesis of purine nucleotides^6^, indicating that it is essential for DNA synthesis^7^. Previous studies have demonstrated that IMPDH was associated with cell growth, malignant transformation and differentiation^8–10^. In mammalian species, it has two ubiquitously expressed isoforms, IMPDH1 and IMPDH2, which are encoded by distinct genes with 85% of their amino acid sequence conserved^11^. IMPDH1 is constitutively expressed in normal cells, whereas expression of IMPDH2 is frequently elevated in malignant cells^12, 13^. Therefore, we focus our study on IMPDH2 as malfunction of IMPDH are primarily due to the up-regulation of this isoform in malignant cells.

Accumulating evidence had demonstrated the involvement of IMPDH2 in different types of malignancies. For instance, enhanced IMPDH2 expression was observed in methotrexate (MTX)-resistant erythroleukemia K562 cells and colorectal cancer cells^14, 15^. The study by Fellenberg *et al*. suggested that IMPDH2 could be used as a promising candidate for the stratification of osteosarcoma patients into low- and high-risk groups^16^. Recently, the protein expression level of IMPDH2 was found to be significantly upregulated in prostate, kidney and bladder cancers and could be a potential biomarker for these diseases^2, 17^. However, to the best of our knowledge, the expression status of IMPDH2 and its clinical significance in NPC remain unknown.

In the current study, immunohistochemistry (IHC) was utilized to examine the expression level of IMPDH2 in NPC tissues and adjacent normal nasopharyngeal tissues. Receiver operating characteristic (ROC) curve analysis was performed to define the cut-off value for separating IMPDH2 expression into increased- and decreased-expression groups. In addition, the relationship between IMPDH2 expression and various clinicopathologic parameters was evaluated to investigate the prognostic impacts of IMPDH2 in NPC patients.

## Results

### IMPDH1 and IMPDH2 expression in NPC cell lines

**Figure 1:**
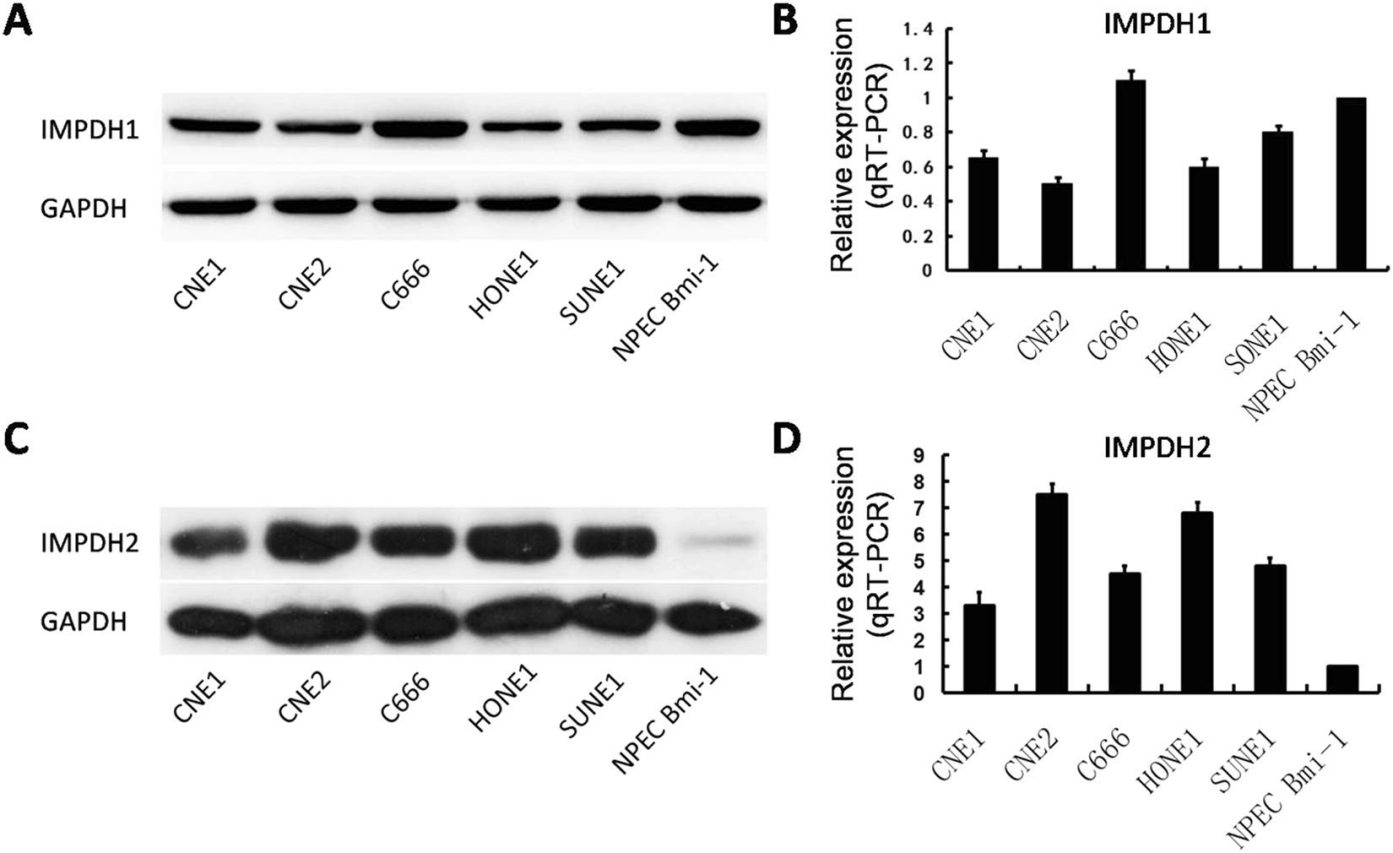
Expression of IMPDH1 and IMPDH2 in NPC cell lines. (**A**) The expression level of IMPDH1 protein in CNE1, CNE2, C666, HONE1, SUNE1 and NPEC2 Bmi-1 was detected by western blotting. (**B**) The expression level of IMPDH1 mRNA in CNE1, CNE2, C666, HONE1, SUNE1 and NPEC2 Bmi-1 was evaluated by quantitative RT-PCR. (**C**) The expression level of IMPDH2 protein in CNE1, CNE2, C666, HONE1, SUNE1 and NPEC2 Bmi-1 was detected by western blotting. (**D**) The expression level of IMPDH2 mRNA in CNE1, CNE2, C666, HONE1, SUNE1 and NPEC2 Bmi-1 was evaluated by quantitative RT-PCR.

Western blotting and qRT-PCR was employed to evaluate the expression levels of IMPDH1 and IMPDH2 protein and mRNA in NPC cell lines including CNE1, CNE2, C666, HONE1, and SUNE1, and an immortalized primary nasopharyngeal epithelial cell line (NPEC2 Bmi-1). As described in Fig. 1A,B, the expression levels of IMPDH1 protein and mRNA in CNE1, CNE2, HONE1, and SUNE1 were lower than that in the NPEC2 Bmi-1. Interestingly, the protein and mRNA levels of IMPDH1 in C666 were higher than that in NPEC2 Bmi-1. However, to our knowledge, the C666 cell line was the only native EBV-infected NPC cell line among these NPC cell lines. These results suggest that high expression of IMPDH1 in C666 may be associated with EBV-infected NPC cell line. By contrast, we performed western blotting and qRT-PCR to assess the protein and mRNA levels of IMPDH2 in NPC cell lines and NPEC2 Bmi-1. As shown in Fig. 1C,D, the expression levels of IMPDH2 protein and mRNA in NPC cell lines were highly expressed, compared to NPEC2 Bmi-1.

### IMPDH2 expression in NPC tissues

**Figure 2:**
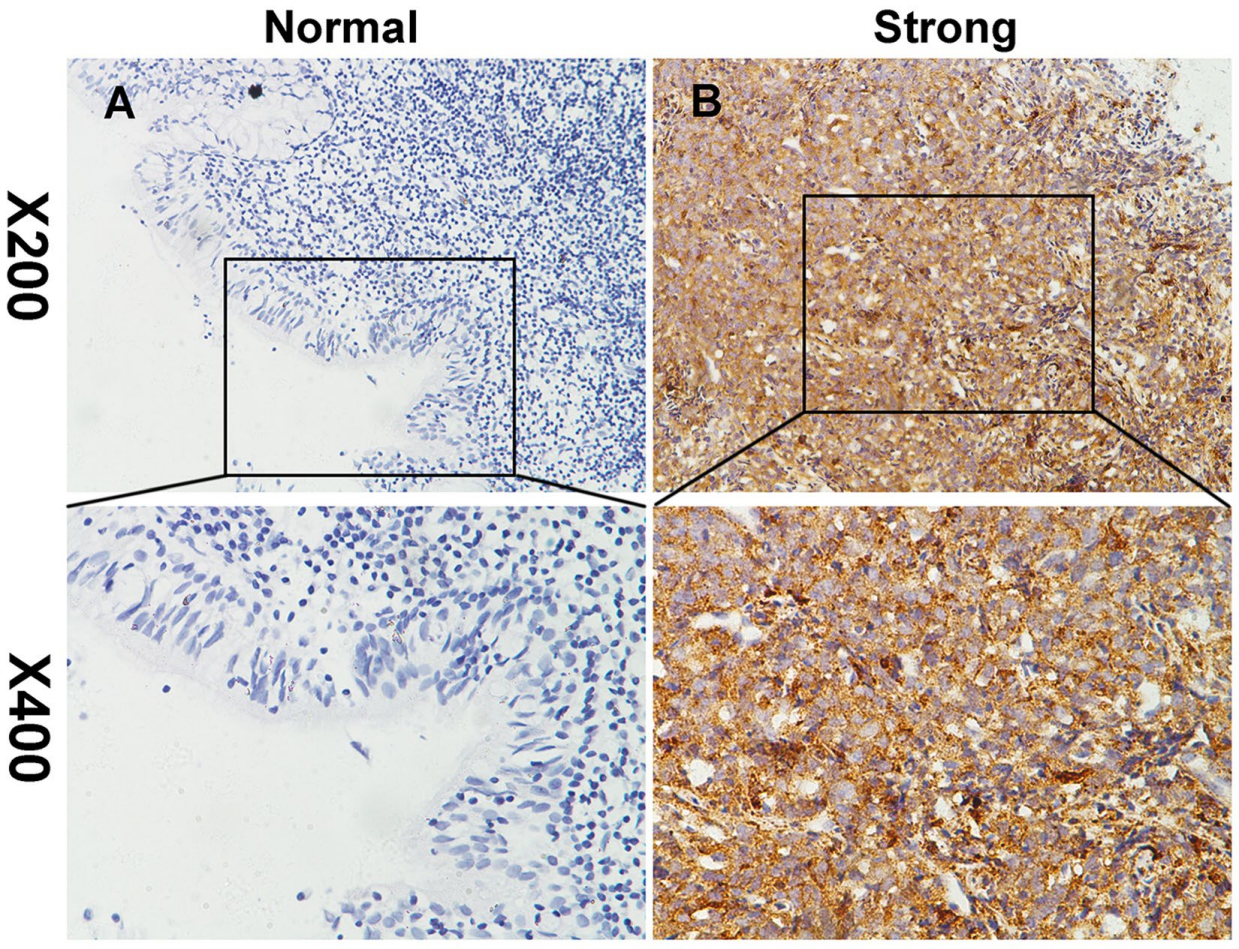
Representative images of IMPDH2 expression in NPC tissues via immunohistochemistry. IMPDH2 was absent or only weakly detected in adjacent normal nasopharyngeal tissues (**A**), whereas its upregulation was mainly detected in NPC tissues (**B**) (original magnification, x200 and x400).

**Figure 3:**
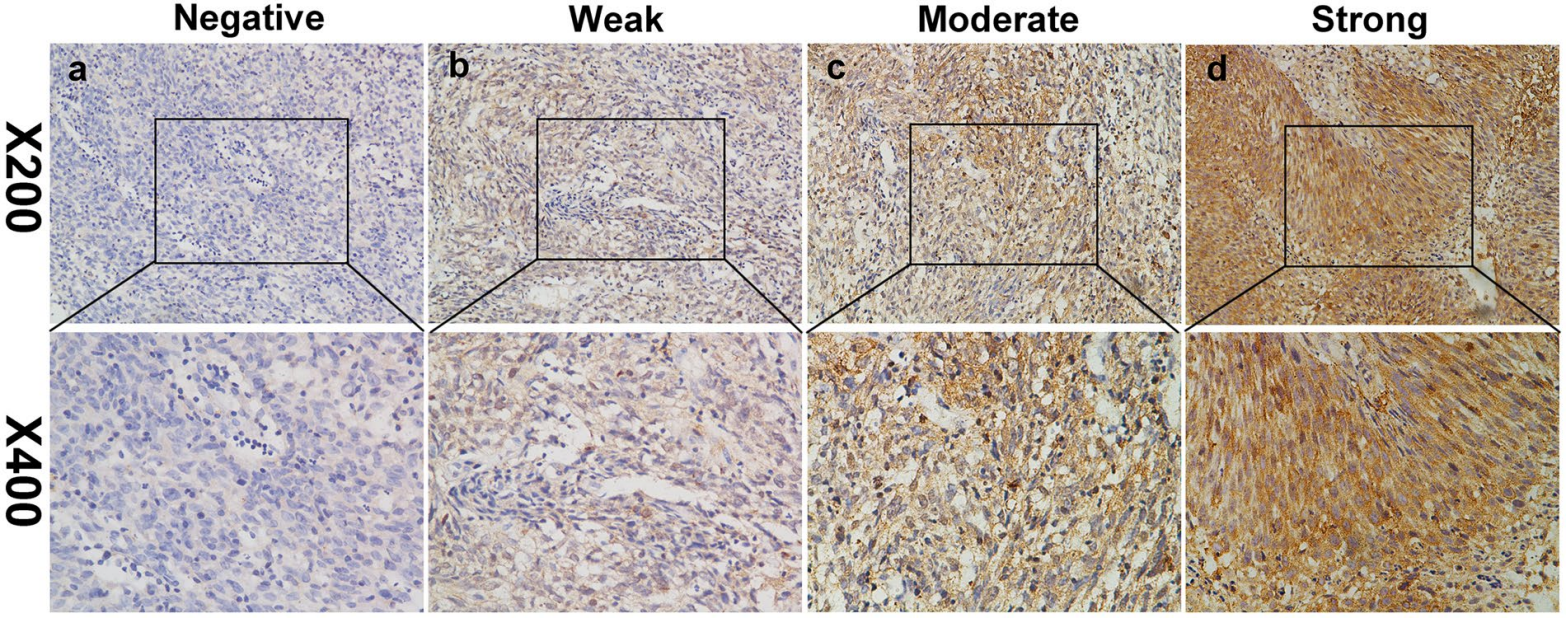
Expression of IMPDH2 in NPC tissues by IHC. The representative images show different staining intensities of IMPDH2: (**a**) negative staining, (**b**) weak staining, (**c**) moderate staining, and (**d**) strong staining (original magnification, x200 and x400).

The expression of IMPDH2 protein was detected by IHC in NPC tissues and adjacent normal nasopharyngeal tissues. Compared with adjacent non-tumorous nasopharyngeal tissues, NPC tissues exhibited higher expression levels of IMPDH2 protein (Fig. 2A,B). The four categories of the intensity of IMPDH2 immunostaining were described in Fig. 3a–d.

### Definition of cutoff score for high IMPDH2 expression in NPC

**Figure 4:**
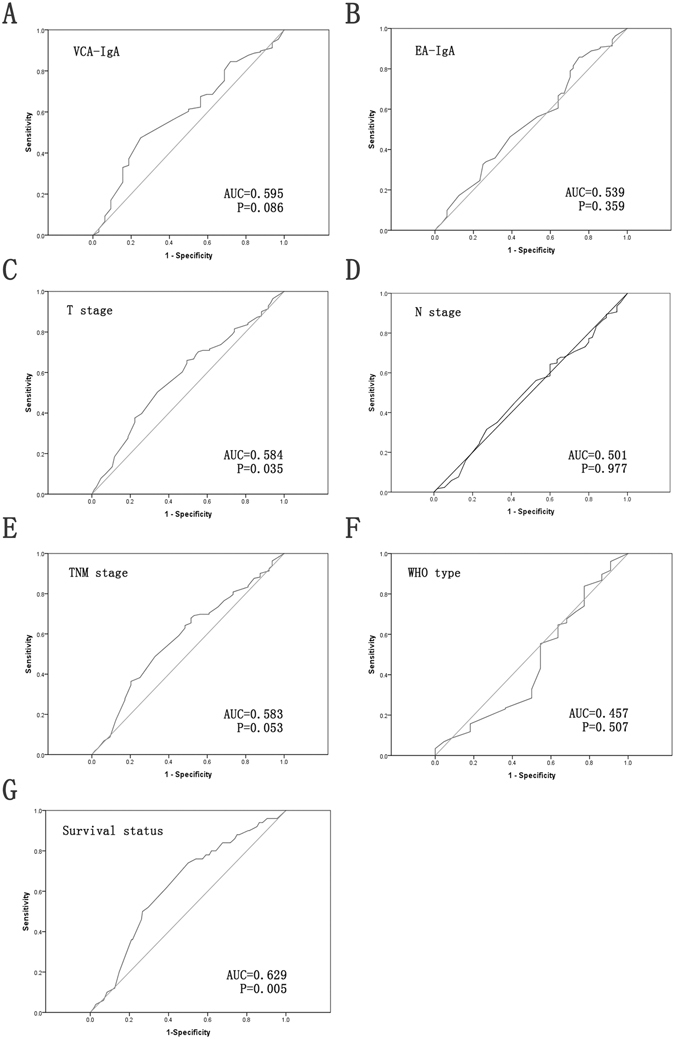
ROC curve analysis was performed to determine the cutoff score for increased IMPDH2 expression. The sensitivity and specificity for each outcome were plotted: VCA-IgA (**A**), EA-IgA (**B**), T classification (**C**), N classification (**D**), TNM stage (**E**), WHO type (**F**) and survival status (**G**).

To better estimate the expression of IMPDH2 in NPC tissues, ROC curve analysis for the clinical features was employed to determine an optimal cutoff value for high IMPDH2 expression. As shown in Fig. 4A–G, ROC curve analysis for survival status had the closest distance from the curve to the point (0.0, 1.0). Therefore, the optimized cut-off value for high IMPDH2 expression was 145. Tumors with scores above this cutoff value were considered to have high IMPDH2 expression, leading to the greatest number of tumors correctly classified, based on the presence or absence of a positive clinical outcome. According to the definition of cutoff score, increased expression of IMPDH2 was observed in 123/226 (54.4%) of NPC tissues.

### The association between IMPDH2 expression and clinicopathological parameters of NPC patients

The chi-square test/Fisher’s exact test was performed to investigate the relationship between IMPDH2 expression and clinicopathological characteristics in 226 cases with NPC (Table 1). Our results suggested significant correlations between IMPDH2 expression and four clinical characteristics including T classification (P = 0.023), TNM stage (P = 0.020), distant metastasis (P = 0.001) and death (P = 0.002). However, we found no statistically significant correlations between IMPDH2 expression and the rest of clinicopathological features, such as age, gender, VCA-IgA, EA-IgA, and WHO type.

**Table 1:** Correlation between IMPDH2 expression and clinical and pathological characters in NPC patients.

Variable	All cases N = 226 (%)	IMPDH2 protein			P value
		Low expression N = 103 (%)	High expression N = 123 (%)	χ^2^	
**Age(years)**				0.054	0.817
≤44	116 (51.3)	52 (44.8)	64 (55.2)		
>44	110 (48.7)	51 (46.4)	59 (53.6)		
**Mean ± SD**(**44.4 ± 11.4**)					
**Gender**				1.097	0.295
Female	52 (23.0)	27 (51.9)	25 (48.1)		
Male	174 (77.0)	76 (43.7)	98 (56.3)		
**VCA-IgA**				1.713	0.191
<1:80	32 (14.2)	18 (56.3)	14 (43.7)		
≥1:80	194 (85.8)	85 (43.8)	109 (56.2)		
**EA-IgA**				0.705	0.401
<1:10	64 (28.3)	32 (50.0)	32 (50.0)		
≥1:10	162 (71.7)	71 (43.8)	91 (56.2)		
**T classification**				5.188	0.023*
T1-2	85 (37.6)	47 (55.3)	38 (44.7)		
T3-4	141 (62.4)	56 (39.7)	85 (60.3)		
**N classification**				0.362	0.547
N0	55 (24.3)	27 (49.1)	28 (50.9)		
N1-3	171 (75.7)	76 (44.4)	95 (55.6)		
**TNM stage**				5.390	0.020*
I-II	64 (28.3)	37 (57.8)	27 (42.2)		
III-IV	162 (71.7)	66 (40.7)	96 (59.3)		
**WHO type**				<0.001	0.990
I + II	22 (9.7)	10 (45.5)	12 (54.5)		
III	204 (90.3)	93 (45.6)	111 (54.4)		
**Distant metastasis**				10.561	0.001*
Yes	61 (27.0)	17 (27.9)	44 (72.1)		
No	165 (73.0)	86 (52.1)	79 (47.9)		
**Death**				9.919	0.002*
Yes	50 (22.1)	13 (26.0)	37 (74.0)		
No	176 (77.9)	90 (51.1)	86 (48.9)		

VCA-IgA: viral capsid antigen immunoglobulin A; EA-IgA: early antigen immunoglobulin A; WHO: World Health Organization; P-values*were calculated using the chi-square test or Fisher’s exact test.

### The relationship of high IMPDH2 expression with poor survival in NPC patients

**Figure 5:**
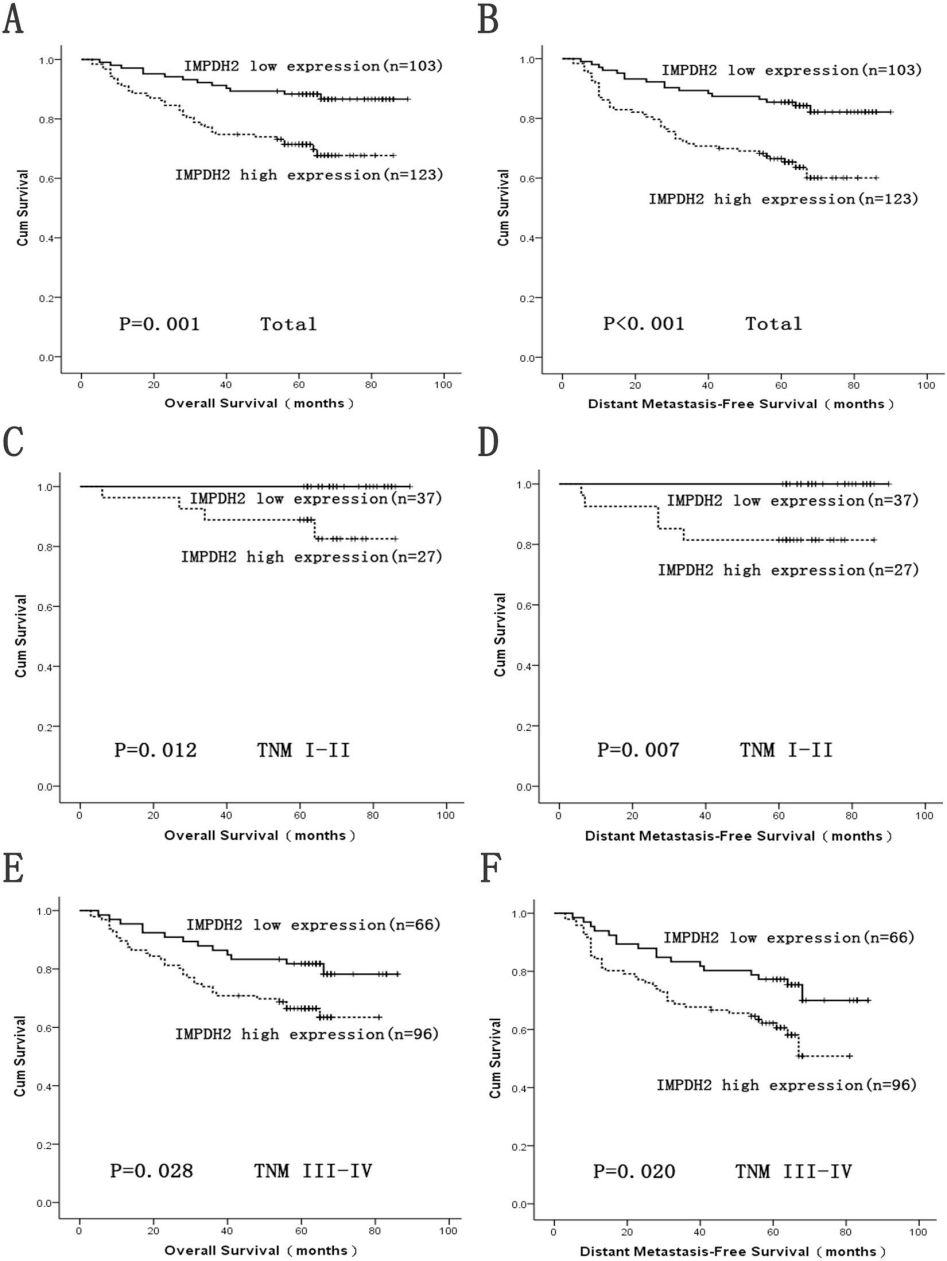
**S**urvival analysis of IMPDH2 expression by Kaplan-Meier method (log rank test). Overall survival (OS) rate and distant metastasis-free survival (DMFS) rate in total (**A**,**B**) NPC patients with low/high IMPDH2 expression. OS rate and DMFS rate in TNM stage I-II (**C**,**D**), TNM stage III-IV (**E**,**F**) NPC patients with low/high IMPDH2 expression.

The relationship between IMPDH2 expression in NPC patients and the survival time of these patients was analyzed by Kaplan-Meier analysis and the log-rank test (Fig. 5A–F). The log-rank test showed that the survival time was different between high and low IMPDH2 expression groups. The median overall survival (OS) time was 52.2 months for the patients with high IMPDH2 expression, significantly shorter than that for the patients exhibiting low IMPDH2 expression (64.4 months) (log-rank test, P = 0.001, Fig. 5A). Furthermore, high IMPDH2 expression group had poorer distant metastasis-free survival (DMFS) time than low IMPDH2 expression group (log-rank test, P < 0.001, Fig. 5B). Moreover, we deployed survival analysis towards the level of IMPDH2 expression in subgroups of NPC patients against TNM stages. Our results revealed that high expression of IMPDH2 could be a prognostic factor for NPC patients with TNM stage I/II (OS: P = 0.012, Fig. 5C; DMFS: P = 0.007, Fig. 5D), TNM stage III/IV (OS: P = 0.028, Fig. 5E; DMFS: P = 0.020, Fig. 5F).

In addition, the prognostic value of IMPDH2 expression was analyzed using univariate analysis model and multivariate Cox regression against OS and DMFS (Table 2). The univariate analysis results confirmed the impact of IMPDH2 expression and TMN stages on survival. The Cox regression further suggested IMPDH2 expression and TMN stages as independent prognostic factors for OS and DMFS in NPC patients. Therefore, the results demonstrated that the IMPDH2 expression level was closely associated with the prognosis of NPC.

**Table 2:** Univariate and multivariate Cox regression analysis of prognostic factors in 226 NPC patients.

Variable	Univariate analysis			Multivariate analysis		
	HR	95% CI	P-value	HR	95% CI	P-value
**Overall survival**						
Age (>44 years vs. ≤44 years)	1.246	0.714–2.173	0.438			
Sex (male vs. female)	0.868	0.462–1.634	0.662			
VCA-IgA (≥1:80 vs. <1:80)	1.043	0.469–2.318	0.918			
EA-IgA (≥1:10 vs. <1:10)	1.083	0.584–2.009	0.799			
WHO type (III vs. I-II)	0.558	0.262–1.189	0.131			
TNM stage (III-IV vs. I-II)	5.415	1.945–15.075	0.001*	4.761	1.704–13.304	0.003*
IMPDH2 expression (High vs. Low)	2.794	1.481–5.269	0.002*	2.428	1.284–4.590	0.006*
**Distant metastasis-free survival**						
Age (>44 years vs. ≤44 years)	1.003	0.607–1.657	0.990			
Sex (male vs. female)	1.035	0.570–1.880	0.911			
VCA-IgA (≥1:80 vs. <1:80)	1.366	0.622–3.003	0.437			
EA-IgA (≥1:10 vs. <1:10)	1.214	0.686–2.149	0.505			
WHO type (III vs. I-II)	0.615	0.303–1.249	0.179			
TNM stage (III-IV vs. I-II)	5.507	2.196–13.811	<0.001*	4.881	1.938–12.295	0.001*
IMPDH2 expression (High vs. Low)	2.654	1.511–4.663	0.001*	2.300	1.306–4.051	0.004*

VCA-IgA: viral capsid antigen immunoglobulin A; EA-IgA: early antigen immunoglobulin A; WHO: World Health Organization; *P-values were calculated using univariate and multivariate cox regression analyses.

## Discussion

Currently, tumor TNM stage is the most commonly and widely used predictive factor for the prognosis of NPC patients. However, the TNM staging alone is inadequate to accurately predict the clinical outcomes of the patients with NPC. Furthermore, the existing molecular markers for NPC have not fully satisfied the clinical prognostic needs. Therefore, it is of great significance to search for novel diagnostic and prognostic markers to refine the individualized treatment of NPC patients.

Accumulating evidence has shown that expression of IMPDH2 was significantly increased in various types of malignancies^12, 13, 18–20^. These findings reveal a potential carcinogenic role of IMPDH2 in multiple human malignancies. To date, however, the expression status of IMPDH2 in NPC and its relationship with the clinicopathological parameters have not been elucidated. In the current study, we first performed immunohistochemistry to evaluate the expression status of IMPDH2 in a cohort of 226 NPC patients. IMPDH2 was significantly up-regulated in NPC paraffin-embedded tissues compared with that in adjacent normal tissues. In particular, overexpression of IMPDH2 protein was detected in 54.4% (123/226) of NPC tissues. By contrast, the expression level of IMPDH2 in the adjacent nasopharyngeal tissues was low or absent. We also observed that IMPDH2 was highly expressed at both the protein and mRNA levels in NPC cell lines compared with NPEC2 Bmi-1. These findings suggest that high expression of IMPDH2 may provide a selective advantage in the NPC tumorigenic processes.

In previous studies, the expression of IMPDH2 was found to be markedly elevated and closely associated with tumor progression and unfavorable prognosis in various different types of malignancies^2, 14, 16, 17^. IMPDH2 was reported to be overexpressed in the subgroup of patients with poor response to chemotherapy, rendering it an independent prognostic factor for chemotherapy response and event-free survival^16^. He *et al*. demonstrated that high IMPDH2 expression was frequently observed in colorectal cancer tissues and might play a crucial role in colorectal carcinogenesis^14^. High IMPDH2 expression might be involved in tumor metastasis and progression in patients with prostate, bladder and kidney cancer^2, 17^. In the present study, we observed that increased expression of IMPDH2 was significantly associated with T classification, TNM stage, distant metastasis and patient death, which suggested that overexpression of IMPDH2 might be involved in the progression of NPC. Additionally, the Kaplan-Meier analysis and log rank test demonstrated that enhanced expression of IMPDH2 significantly correlated with shorter OS and poorer DMFS in patients with NPC. Further stratified analysis revealed that elevated expression of IMPDH2 could predict the unfavorable outcome in NPC patients with TNM stage I/II and III/IV. More importantly, multivariate Cox regression analysis showed that high IMPDH2 expression was identified as an independent prognostic factor for OS and DMFS in NPC. Hence, our study suggested the potential use of IMPDH2 expression in addition to existing classifications to predict OS and DMFS in patients with NPC.

With respect to the function of the IMPDH gene, as a rate-limiting enzyme in the de novo guanine nucleotide biosynthesis, IMPDH has been implicated in cell proliferation, differentiation and the development of chemoresistance. Inhibition of IMPDH contributed to depletion of guanine nucleotide pools, followed by decreased synthesis of DNA and RNA^21^. A study by Takebe N *et al*. demonstrated that inhibition of IMPDH had been able to induce caspase-dependent apoptosis and cell-cycle arrest in multiple myeloma cells^22^. Furthermore, IMPDH inhibitors, such as tiazofurin and benzamide riboside, has been found to induce terminal differentiation in several types of human cancer cells^23, 24^ and has been successfully applied in clinical trials^25, 26^. On the other hand, overexpression of IMPDH2 induced a strong chemoresistance in osteosarcoma cells which was mediated partly by upregulation of anti-apoptotic proteins, leading to inhibition of the mitochondrial apoptotic signaling pathway^27^. In addition, researchers found the sensitivity of HT29 human colon cancer cells were significantly increased towards chemotherapy by down-regulating the IMPDH2 gene expression in the resistant cells^28^. As a consequence, IMPDH2 gene expression by more effective inhibitors in combination with chemotherapy may have the potential to provide an effective treatment to overcome chemoresistance.

In summary, we revealed for the first time that IMPDH2 protein was markedly expressed in NPC, and elevated expression of IMPDH2 was closely associated with unfavorable prognosis in NPC. These data suggested that the expression levels of IMPDH2 protein, as detected by IHC, could be identified as a prognostic biomarker for the clinical outcome of NPC patients and a potential therapeutic target in NPC. However, further investigation is needed to elucidate the molecular mechanisms on the oncogenic function of IMPDH2 for NPC.

## Materials and Methods

### Patients and tissue specimens

Paraffin-embedded specimens from 226 patients with primary NPC were obtained from Sun Yat-Sen University Cancer Center between 2001 and 2004. These patients’ clinical and pathological data, such as age, gender, VCA-IgA, EA-IgA, T classification, N classification, TNM stage and WHO type, were also included in this study (Table 1). None of these patients had previous malignant disease, second primary tumor, any history of radiotherapy, chemotherapy or surgical treatment. The 226 patients included 174 males (77.0%) and 52 females (23%) with ages ranging from 15 to 77 years (mean, 44 years). The tumor stages were defined according to the seventh edition of the American Joint Committee on Cancer Staging manual. All patients were treated with conventional two-dimensional radiotherapy with or without chemotherapy. The follow-up data were obtained by telephone or from the outpatient records. Overall survival (OS) was defined as the time from the date of diagnosis to the date of NPC-related death. Distant metastasis-free survival (DMFS) was defined as the interval from the date of diagnosis to the date of distant metastasis/metastases. The study was approved by the Institutional Research Ethics Committee of Sun Yat-Sen University Cancer Center and written informed consent was obtained from all subjects. All experimental methods were carried out in accordance with approved guidelines of Sun Yat-Sen University Cancer Center.

### Immunohistochemistry

Immunohistochemical analysis was carried out to examine the IMPDH2 expression levels with a standard two-step method in 226 NPC tissue specimens. The paraffin-embedded NPC specimens were dried at 65 °C for 2 h. Subsequently, the slides were deparaffinised in xylene, rehydrated through a series of graded alcohol and immersed in 3% hydrogen peroxide for 15 min to block the endogenous peroxidase activity. Then the slides were boiled in citrate antigen retrieval solution (pH 6.5) for 20 min in a microwave oven for antigen retrieval. After washing three times for 5 min in phosphate buffered saline (PBS), the sections were incubated with a primary antibody against IMPDH2 (1:800 dilution; Abcam Antibodies, Epitomics, U.S.A.) at 4 °C overnight. After rinsing three times, the tissue sections were sequentially incubated with a secondary antibody for 1 hour at room temperature. After three further washes in PBS, the sections were stained with 3,3-diaminobenzidine (DAB), counterstained with Mayer’s haematoxylin, dehydrated and mounted. A negative control was obtained by replacing the primary antibody with PBS. Known immunostaining-positive slides were used as the positive controls.

### IHC evaluation

Protein expression levels of IMPDH2 were evaluated by Semi-quantitative IHC detection. A brown particle in cytoplasm was considered as positive labeling. In brief, the staining intensity was classified as 0 (no staining), 1+ (weak), 2+ (moderate) or 3+ (strong) (I0, I1–3). Then, the proportion of tumor cells with that intensity was divided by the total number of tumor cells and recorded in 5% increments from 0 to 100 (P0, P1–3). Each IHC score was calculated based on the staining intensity and the proportion of the area stained according to the equation: IHC score = I1 × P1 + I2 × P2 + I3 × P3 (range 0–300). The evaluation of immunohistochemical results was performed by three independent pathologists who were blinded to the clinicopathological data. The same methods were applied for both tumorous and adjacent non-tumorous specimens.

### Selection of cutoff score

Receiver operating characteristic (ROC) curve analysis was applied to determine a cutoff score for IMPDH2 expression, by using a 0, 1- criterion. Briefly, the sensitivity and specificity for the evaluated outcome were plotted to generate various ROC curves. The score with the shortest distance from the curve to the point (i.e., 0.0, 1.0) at both maximum sensitivity and specificity was selected as the cutoff score to determine the greatest number of tumors which were correctly classified as having or not having the clinical outcome. For the purpose of the ROC curve analysis, the clinicopathologic parameters were dichotomised as follows: VCA-IgA (<1:80 vs. ≥1:80), EA-IgA (<1:10 vs. ≥1:10), T stage (T1 + T2 vs. T3 + T4), N stage (N0 vs. N1 + N2 + N3), TNM stage (I + II vs. III + IV), WHO type (I + II vs. III) and survival status [NPC-related death vs. others (censored, alive or death from other causes)].

### Cell lines and cell cultures

The immortalized nasopharyngeal epithelial cells (NPECs) induced by Bmi-1 (NPEC2 Bmi-1) were established and grown in keratinocyte/serum-free medium (Invitrogen). The NPC cell lines CNE1, CNE2, C666, HONE1 and SUNE1 were maintained in RPMI-1640 medium supplemented with 10% fetal bovine serum (FBS) (Gibco, USA). The cell lines were cultured in a humidified incubator at 37 °C with 5% CO_2_.

### Western blotting

Equal amounts of whole-cell lysates were separated by SDS-polyacrylamide gel electrophoresis (PAGE) and subsequently transferred onto a polyvinylidene difluoride (PVDF) membrane (Pall Corp., Port Washington, NY). The membrane was blocked with 5% skimmed milk and then incubated with primary mouse monoclonal antibodies against IMPDH2 (1:1000 dilution, Abcam Co Ltd, USA) and GAPDH (1:1000 dilution; Abcam, Cambridge, MA, USA), respectively. After thoroughly washing the membrane, it was then incubated with the secondary anti-mouse antibody from Santa Cruz Biotechnology (Santa Cruz Biotechnology, CA, USA). Finally, the immunoreactive proteins were detected by an enhanced chemiluminescence (ECL) western blotting detection protocol.

### Quantitative real-time polymerase chain reaction (qRT-PCR)

Total RNA was extracted from the NPC cell lines using Trizol regent (Invitrogen, Grand Island, NY, USA) and cDNA was synthesized by SuperScript *Reverse Transcriptase* kit (Promega, Madison, WI, USA) according to the manufacturer’s instruction. The primer sequences used to amplify IMPDH2 were: 5′-GCT CCT GTG CCT GAT GGA AT-3′ and 5′-CGG GCT CCT CCC CAA AAT AA-3′. GAPDH was used as an internal control for normalization.

### Statistical analysis

Statistical analyses were performed with SPSS 19.0 (IBM). P values less than 0.05 were considered to be statistically significant. Overall survival (OS) was defined as the interval between surgery and death or between surgery and the last observation. Distant metastasis-free survival (DMFS) was defined as the time from surgery to distant metastasis or death due to NPC. The Chi-square test or Fisher’s exact test was performed to evaluate the correlation between IMPDH2 expression and the clinicopathological variables. The cumulative survival distributions were estimated using the Kaplan-Meier analysis and the log-rank test. The significance of various clinicopathological characteristics was evaluated using univariate Cox regression model. These statistically significant variables based on the univariate analysis were selected for reassessment in the multivariate Cox proportional hazard regression model.

## Electronic supplementary material

Supplementary information
