# Supplementary material for: High expression of IMPDH2 is associated with aggressive features and poor prognosis of primary nasopharyngeal carcinoma
Source: Sci Rep. 2017 Apr 7;7:745. doi: 10.1038/s41598-017-00887-1 (PMC5429725; doi:10.1038/s41598-017-00887-1)

High expression of IMPDH2 is associated with aggressive features and poor prognosis of primary nasopharyngeal carcinoma

Yi Xu[1,2]*, Zhousan Zheng[1]*, Ying Gao[1]*, Shiyu Duan[3,4], Cui Chen[1], Jian Rong[5], Kebing Wang[6], Miao Yun[2,7], Huiwen Weng[1], Sheng Ye[1] & Jiaxing Zhang[1,2]

[1]Department of Oncology, The First Affiliated Hospital, Sun Yat-Sen University, Guangzhou 510080, China. [2]Sun Yat-sen University Cancer Center, State Key Laboratory of Oncology in South China, Collaborative Innovation Center for Cancer Medicine, Guangzhou 510060, China. [3]Department of Pathology, Nanfang Hospital, Southern Medical University, Guangzhou 510515, China. [4]Department of Pathology, School of Basic Medical Sciences, Southern Medical University, Guangzhou 510515, China. [5]Department of Extracorporeal Circulation, The First Affiliated Hospital, Sun Yat-sen University, Guangzhou 510080, China. [6]Department of Surgical Laboratory, The First Affiliated Hospital, Sun Yat-Sen University, Guangzhou 510080, China. [7]Department of Ultrasound, Cancer Center, Sun Yat-Sen University, Guangzhou 510060, China.

Correspondence and requests for materials should be addressed to S.Y. (yes20111212@163.com) or J.X.Z. (zjxlundy@hotmail.com)

*Yi Xu, Zhousan Zheng and Ying Gao contributed equally to this work.

Supplementary figure legends

Supplementary Figure S1. IMPDH1 expression in NPC cell lines and NPEC2 Bmi-1 by Western blotting and qRT-PCR.

The expression levels of IMPDH1 protein and mRNA in CNE1, CNE2, HONE1, and SUNE1 were lower than that in the NPEC2 Bmi-1**(Supplementary Figure S1A-B)**, whereas the protein and mRNA levels of IMPDH1 in C666 were higher than that in NPEC2 Bmi-1**(Supplementary Figure S1A-B)**.

Supplementary Figure S1

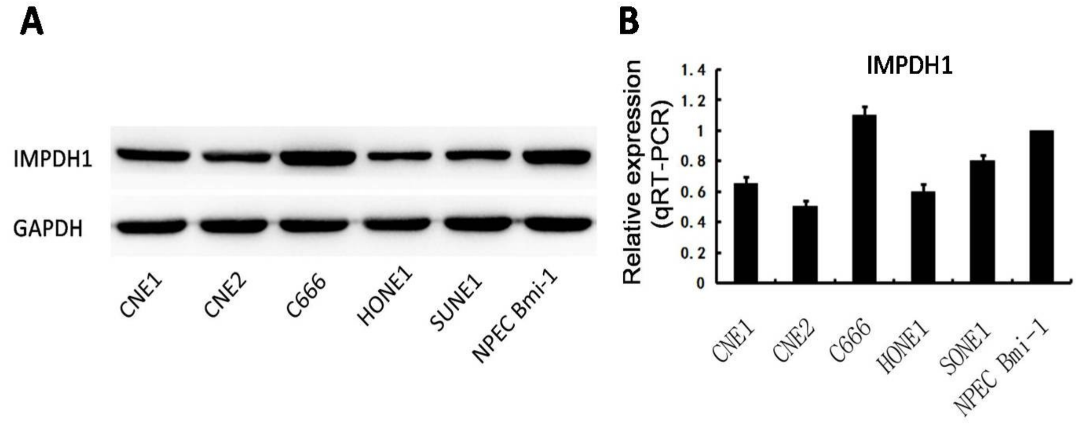

Supplement: Supplementary file 1 — Supplementary information [file 41598_2017_887_MOESM1_ESM.pdf]
